# Probiotic Chocolate Containing *Lactobacillus plantarum* Dad-13 Alters the Gut Microbiota Composition of Undernourished Children in Lombok: A Randomized Double-Blind Trial

**DOI:** 10.1155/2024/9493797

**Published:** 2024-08-03

**Authors:** Endang S. Rahayu, Wahyu K. Yoga, Husnita Komalasari, Mariyatun Mariyatun, Wednes A. Yuda, Nancy E. P. Manurung, Pratama N. Hasan, Suharman Suharman, Fathyah H. Pamungkaningtyas, Dina A. Nurfiana, Putrika C. Pramesi, Mifta Gatya, Phatthanaphong Therdtatha, Jiro Nakayama, Mohammad Juffrie, Titiek F. Djaafar, Tri Marwati, Tyas Utami

**Affiliations:** ^1^ Faculty of Agricultural Technology Universitas Gadjah Mada, Yogyakarta 55281, Indonesia; ^2^ Center for Food and Nutrition Studies Universitas Gadjah Mada, Yogyakarta 55281, Indonesia; ^3^ Chocolate Factory “nDalem”, Yogyakarta 55261, Indonesia; ^4^ Food Technology Study Program Department of Chemical Engineering Politeknik Negeri Sriwijaya, Palembang 30128, Indonesia; ^5^ School of Agro-Industry Faculty of Agro-Industry Chiang Mai University, Chiang Mai 50100, Thailand; ^6^ Department of Bioscience and Biotechnology Faculty of Agriculture Kyushu University, Fukuoka 819-0395, Japan; ^7^ Department of Public Health Universitas Gadjah Mada, Yogyakarta 55281, Indonesia; ^8^ Research Center for Food Technology and Process National Research and Innovation Agency, Yogyakarta 55861, Indonesia

## Abstract

The present study investigated the ingestion effect of chocolate probiotic containing *Lactobacillus plantarum* Dad-13 in undernourished children. A 100-day observation was conducted on undernourished children in Lombok, who were divided into probiotic (*n* = 28) and placebo (*n* = 28) groups. Fecal sampling was performed on the 10^th^ and 100^th^ days and further analyzed for gut microbiota composition, short-chain fatty acid (SCFA), and fecal pH. A significant difference was found in the diversity index, fecal pH, and several microbiotas at the phylum and genus levels. At the phylum level, *Bacteroidetes* was significantly higher in the probiotic group, and a higher relative abundance (RA) of *Firmicutes* was found in the placebo group. At the genus level, significant differences were observed in some bacteria, such as *Bifidobacterium* and *Prevotella*. Therefore, it can be concluded that the probiotic intervention in this study resulted in changes of gut microbiota diversity and fecal pH.

**Trial Registration:** Thai Clinical Trials Registry identifier: TCTR20220425001

## 1. Introduction

Malnutrition is a major health problem for every country in the world. According to the World Health Organization (WHO) Indonesia [1], malnutrition is a condition of deficiency, excess, or imbalance of a person's nutritional intake, which includes excess or malnutrition. Malnutrition, especially the malnutrition that affects children under five, will affect children's growth and development in the future if not handled properly. Indonesia is one of the developing countries with a high percentage of malnourished sufferers. West Nusa Tenggara is one of the provinces in Indonesia with a percentage of poor nutritional status. In 2022, West Nusa Tenggara was ranked third for the Indonesian region with the most stunting cases in children under 5 years old with a prevalence of 32.7% [2]. In the same year, the Indonesian Central Statistical Agency reported that 23,791 babies were born malnourished (5.3%) in West Nusa Tenggara [3].

Malnutrition is a condition where the body does not obtain adequate nutrition or an imbalance between the food consumed and the nutritional needs for growth, development, and maintaining health. According to Monira et al. [4], children with malnutrition experience changes in the composition of the gut microbiota; thus, they are more likely to experience gastrointestinal infections. This finding is supported by that of Hashimoto et al. [5], who stated that malnutrition causes dysbiosis or changes in the composition of the gut microbiota in the patient's intestine. The gut microbiota of children with malnutrition or undernourished has significantly lower microbiota diversity and a lower number of *Bacteroidetes* and pathogenic *Proteobacteria* and *Firmicutes* than normal or healthy children [4, 6–8]. In addition, based on a research in Malawi, the abundances of *Prevotella*, *Bacteroidetes*, *Eubacterium,* and *Blautia* species were lower in malnourished children than in undernourished children [9].

The gut microbiota can be related positively or negatively in influencing the absorption of nutrients in the gut. Microbiota contributes to the proliferation and maturation of intestinal epithelial cells, induction of host genes for nutrient absorption, development of the mucosal immune system which is important for optimal absorption of nutrients, and regulation of energy metabolism through the digestion of indigestible polysaccharides which are then fermented by microbiota to produce short-chain fatty acids (SCFAs) [10]. A study in Yogyakarta, Indonesia, investigated the difference in gut microbiota and SCFAs between normal and moderately malnourished children. The study reported that the levels of propionic acid and butyric acid were higher in normal compared with undernourished subjects [11]. SCFAs influence the colonic environment, ensuring that nutrients are properly absorbed. SCFAs have specific benefits for each component: acetic acid has the highest concentration and functions as the main substrate for cholesterol synthesis, and propionic acid is an antimicrobial and anti-inflammatory and increases insulin sensitivity; butyric acid is anticarcinogenic and anti-inflammatory and can be useful for preventing colon cancer [12].

Various studies have been conducted to improve the nutritional status of malnourished children, one of which is through the consumption of probiotics. Probiotics are live bacteria that can have health effects on their host if consumed in sufficient quantities [13]. Probiotics can maintain the balance of the intestinal microbiota, increase body immunity, and benefit other body health [14]. The consumption of probiotics by malnourished children also reduces diarrhea morbidity [15]. The research on local probiotics and their application to chocolate products has never been carried out.

This study used a local probiotic strain, namely, 10^9^ CFU/g *Lactobacillus plantarum* Dad-13, to be applied to a chocolate bar, hereinafter referred to as probiotic chocolate. Chocolate is a food product favored by children and a good carrier of probiotics [16]. The research of Rahayu et al. [15] showed that indigenous probiotic strains, including *Lactobacillus plantarum* Dad-13, that have been confirmed molecularly are resistant to pH 2 and bile salts at a concentration of 3% and can inhibit the growth of pathogenic bacteria. These bacteria have also been tested safe for consumption and can grow and colonize the digestive tract; thus, their ability as probiotics has been tested [17]. The study proposes that the ingestion of chocolate containing the indigenous probiotic strain, *Lactobacillus plantarum* Dad-13, will result in advantageous health outcomes for the participants. It is expected that the probiotic component of the chocolate will change the composition of the gut microbiota in undernourished children in Lombok, resulting in better nutritional results and general health.

## 2. Materials and Methods

### 2.1. Study Population

The subjects recruited for this study went through several stages, including data gathering, screening, and grouping of prospective subjects. A sampling size was set to determine the number of subjects. The subjects were children aged 8–12 years from SDN 3 and 5 Belanting, East Lombok. A total of 133 children participated in the screening process. The selection of subjects was performed using anthropometric measurement, which was used to describe their nutrition status. From the measurement, 61 children were healthy or normal, 63 were undernourished, and 9 were malnourished. The eligibility criterion for the subjects was the *z*-score between −3 to <–2 standard deviation (SD) based on body mass index (BMI)/age. A total of 63 children passed the eligibility screening to join the research. However, only 56 of the subjects completed the research.

Demographic data, including age, gender, education and occupation of parents, and the children's weight and height, were collected. Children's height was measured using a microtoize (0.1 cm accuracy), and a digital scale was used to measure their weight. The participants must be residents of Belanting, Lombok, Timur, can read, understand, and agree with research provisions, do not have a history of allergy to the components of probiotic products or placebo, are physically and mentally healthy, do not consume antibiotics and probiotics other than the chocolate products provided, and do not drink alcohol and smoke. Participants were not allowed to participate in the study if they are consuming alcohol; have digestive disorders; are taking antibiotics, supplements, or immunoregulators; and have underwent gastrointestinal surgery. A subject was forced to discontinue the observation if their fecal samples were not collected on a predetermined day, they did not fill in the recruitment data, failed to meet the consumption dose of chocolate products during the study, and took antibiotics or probiotics other than the research sample.

### 2.2. Ethical Approval and Protocol Registry

The study protocol was approved by the Medical and Health Research Ethics Committee (MHREC), Faculty of Medicine, Universitas Gadjah Mada (UGM) (Approval reference: KE/FK/0872/EC/2019) on July 26, 2019. The protocol of this study was registered. This research was conducted in alignment with regulations that are applicable in Indonesia (approved on April 25, 2022) and based on the guidelines from the World Medical Assembly (Declaration Helsinki, last amendment, Edinburgh, Scotland, 2000 and last clarified in Tokyo, 2004) with notes of CPMP on GCP (CPMP/ICH/135/95). Informed consents were signed with the parent/legal guardian before the observation.

### 2.3. Sample Size Determination

The sample size was calculated based on a two-sided test (Equation (1)) using the software sample sizer based on “Sample Size Determination in Health Studies: A Practical Manual” from the WHO published in 1991 by Lwanga and Lemeshow [18]:

(1) $$\begin{array}{c} n=\frac{2\;\delta^{2}{\left(Z1-\alpha /2+Z1-\beta \right)}^{2}}{{\left(\mu 1-\mu 2\right)}^{2}} \end{array}$$

The sample size (*n*) was calculated using the SD of the population (*δ*), assumed 0.95 as the significance level (*α*), the mean of the intervention group's BMI (*μ*1), and the mean of the control group's BMI (*μ*2). Both means of the group, with values of 22.46 ± 3.35 and 2368375kg/m^2^, were referred to the work of Pan et al. [19]. The minimum number of subjects obtained from the number of sample size after multiplication with the correction factor (lost due to follow-up) was 20. A correction factor was used to anticipate the subject who would drop out during observation. Through calculations, the minimum number of subjects for observation was three people.

### 2.4. Research Products

The research product was a probiotic chocolate containing chocolate and an indigenous probiotic *L. plantarum* Dad-13 (the whole genome sequencing (WGS) data is available in GenBank database; accession number: JALLKE000000000) (1 × 10^9^ CFU/bar) for the probiotic group, whereas the placebo group was given chocolate with the same composition without probiotics. The product was prepared in the form of a chocolate bar, which was consumed once a day during observation (90 days). The probiotic *L. plantarum* Dad-13 was deposited in ampoules at the Food and Nutrition Culture Collection, Center for Food and Nutrition Studies, UGM, Indonesia. Subsequently, the probiotic powder was prepared using halal media. After biomass production followed by a freeze drying process, the probiotic powder was integrated into a chocolate bar and stored at a refrigerator (< 4°C) before consumption. The chocolate probiotic powder (5 g) was distributed to the subjects according to the treatment groups.

### 2.5. Research Design

This research was conducted using a randomized, double-blinded, placebo-controlled trial. An independent party from nDalem chocolate factory (Yogyakarta, Indonesia) generated a random allocation sequence using block randomization with the 2-block permutation method stratified by subject type (probiotic and placebo groups). Meanwhile, the researchers were blinded by the group that consumed the placebo or product, as the groups were encoded with three random numerical digits. The third party concealed the allocation sequence and dispensed the blinded investigational product accordingly. Before distribution to the subjects, the products were prepared by an independent technician from the Center for Food and Nutrition Studies, UGM.

In total, the study was performed for 100 days (June–October 2019). During the first 10 days, the selected subjects were asked not to consume any antibiotics or probiotic products (baseline period). Then, the subjects consumed the research products based on the group they were assigned to for the remaining 90 days. Fresh fecal samples were collected on the 10th and 100th day. The fecal samples were then analyzed for pH, SCFAs, and gut microbiota.

### 2.6. Fecal Sample Collection and DNA Extraction

Fecal samples were collected twice on the 10th and 100th day in sterile tubes containing 2 mL RNA later (Sigma-Aldrich; Saint Louis, USA) as DNA/RNA stabilizers. Subsequently, the fecal samples were transported to the laboratory in a cooler box (< 10°C) within a maximum of 1 h after collection. The fecal samples were labeled and stored in a freezer at a temperature of −25°C for further molecular analysis.

DNA extraction was performed in accordance with the work of Nakayama [20] but with modifications. First, the sample was prepared by 10-fold dilution with RNA later and continuously washed with phosphate-buffered saline. Then, the sample solution was added with 300 *μ*L Tris-sodium dodecyl sulfate solution, mixed, and resuspended. The suspended solution was added with 500 *μ*L TE buffer-saturated phenol and placed in a bead beater (Bead Mill Homogenizer, Benchmark Scientific, China) at 40000 rpm for 60 s. The resulting supernatant and 200 *μ*L phenol/chloroform/isoamyl alcohol (25 : 24 : 1; *v*/*v*) (Sigma-Aldrich; Saint Louis, USA) went through another cycle in the bead beater at 4000 rpm for 90 s. Afterward, centrifugation was conducted at 13,000 rpm for 5 min at 4°C. The obtained supernatant was mixed with 25 *μ*L 3 M sodium acetate (pH 5.2) (Sigma-Aldrich; Saint Louis, USA) and 300 *μ*L isopropanol and incubated for 30 min at −30°C. Another centrifugation was conducted at 13,000 rpm for 5 min at 4°C. The DNA pellet was washed with 500 *μ*L ice-cold 70% ethanol and then centrifuged at 13,000 rpm for 5 min at 4°C. The DNA pellet was air-dried at room temperature. The pellet was suspended in 20 *μ*L TE buffer (pH 8.0) and stored at −30°C until use.

### 2.7. Gut Microbiota Analysis

The gut microbiota composition of the fecal samples from subjects was analyzed as the primary outcome measure. DNA was analyzed using 16S rRNA sequencing, referring to Nakayama's protocol. The target for sequencing was the V3–V4 region (F (Bakt_341F): CGCTCTTCCGATCTCTGCCTACGGGNGGG-WGCAG, R (Bakt_805R): TGCTCTTCCGATCTGACGACTACHVGGGTATCTAATCC). Afterward, using the MiSeq v3 chemistry, the secondary polymerase chain reaction (PCR) products were subjected to paired-end sequencing. The sequence data processing for the construction of operational taxonomic units (OTUs) with 97% identity and removal of PCR chimeras was conducted using Usearch [21, 22]. Each OTU taxonomy was analyzed using the SINTAX command and Greengene database with a cut-off value of 0.8. The downstream analysis was performed using quantitative insight into microbial ecology.

### 2.8. Fecal pH Analysis

The results of fecal pH analysis were determined as secondary outcome measures. The fecal pH was determined on the 10th and 100th day. Each fecal sample was weighed at 0.2 g and added with 1 ml distilled water prior to homogenization using vortex. pH was measured by inserting the glass electrode of a pH meter [23].

### 2.9. SCFA Analysis

Alongside the fecal pH analysis, the results of fecal SCFA analysis were considered secondary outcome measures. The analysis of SCFAs was performed on the first and 61st days according to the work of Salazar et al. [24]. One gram of fecal sample was added to distilled water at a ratio of 1 : 3. The mixture was homogenized using a vortex for 5 min, followed by centrifugation at 10000 rpm for 10 min. The supernatant was then analyzed using gas chromatography (Shimadzu, GC 2010 plus series) with specifications of 240°C injector, RTX-Wax column, column length of 30 m, column temperature of 145°C, diameter of 0.25 mm, and column flow of 0.8 min, with helium as the carrier gas and flame ionization detector operating at 240°C.

### 2.10. Statistical Analysis

Statistical analysis was performed using IBM Statistic SPSS 25.0 with 95% and 99% confidence intervals. Demographic and baseline characteristics were calculated using descriptive statistics for both the total subjects and each group. In addition, to assess the homogeneity of baseline characteristics between groups, we used the Pearson chi-square analysis for categorical data and independent *t*-tests or Mann–Whitney tests for numerical data, depending on the fulfilment of the normal distribution assumption. Paired *t*-tests and Wilcoxon tests were used to analyze the observed parameters during intervention, depending on the distribution assumption. For the gut microbiota composition, statistical analysis was conducted using R (v.4.0.3) in R studio (v. 1.1.463) with ggplot2 (v. 3.3.2) and vegan (v.2.5-6) packages. Wilcoxon rank-sum test was used for comparison between groups, and permutational multivariate analysis of variance was used to evaluate the gut microbiota composition at the OTU and genus levels. In addition, nonmetric multidimensional scaling (NMDS) based on the Bray–Curtis dissimilarity was used to visualize the difference between groups. The statistical analysis of gut microbiota composition using the online version of linear discriminant analysis effect size (LEfSe) was completed online for visualization of the taxonomy profile. Linear discriminant analysis (LDA) was performed using one-against-all criteria, and the threshold on the logarithmic was 2. The alpha value of the Kruskal–Wallis and pairwise Wilcoxon tests was 0.05.

## 3. Results and Discussion

### 3.1. Participant Flow

**Figure 1:**
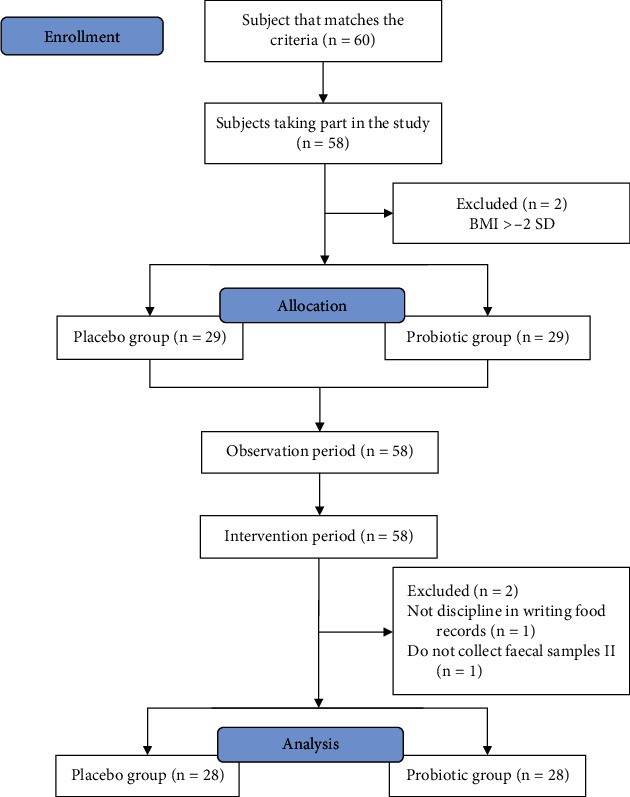
CONSORT participant flow.

Figure 1 shows the enrolment of the study. On day 0 of the study, as many as 60 selected subjects were remeasured for their body weight and height.

Since the screening period up to the H-1 study, two subjects had to be excluded from the study because they had a *z*-score of −3 to <−2 SD based on BMI/age. Thus, 58 subjects were followed up in the randomization phase, with each of the placebo and probiotic groups consisting of 29 people until the end of the intervention period. At the end of the intervention period, two subjects were excluded because they met the discontinuity criteria, namely, one subject failed in keeping food records, and one subject was unsuccesful in collecting the second fecal sample. The final number of subjects who participated in the research was 56 subjects.

### 3.2. Subject Characteristics

The subjects comprised 56 children aged 8–12 years and were divided into two groups, with each group consisting of 28 subjects. The mean and SD of body weight and height for the placebo group were 23.42 ± 2.88 and 125.79 ± 5.96, respectively. The values were 23.53 ± 2.71 (body weight) and 126.78 ± 5.69 (height) for the probiotic group. The subjects had BMIs of 14.75 ± 0.88 and 14.62 ± 0.99 for the placebo and probiotic group, respectively, with a *z*-score of −3 to <−2 SD based on BMI/age, which categorized as undernourished or underweight children. Thus, the subjects who participated in this study were children with poor nutritional status. Based on Table 1, the nutritional intake of undernourished children during screening was 4.57 ± 1.88 times/day in the probiotic group and 4.86 ± 1.92 times/day in the placebo group.

### 3.3. Nutrient Intake of Subjects During the Intervention Period

The nutrition intake of undernourished subjects during the intervention period was analyzed based on food record data recorded for 90 days (Table 2).

Table 2 shows that the mean intake of macro and micronutrients in both groups still did not meet the recommended RDA standards. Significant differences were observed between groups in terms of dietary fiber, folic acid, and magnesium.

The 2010 Dietary Guidelines for Americans Advisory Committee report stated that the main role of carbohydrates is to provide energy for all cells in the body and dietary fiber intake [25]. Protein is a basic nutrient that is useful in the formation of protoplasma cells; it must be consumed in sufficient quantities because it is very important in the process of growth, and vitamins and minerals must be available in sufficient quantities. Riskesdas in 2013 stated that the energy intake of < 70% implies the lack of energy intake; the subjects in this study had an energy intake of < 50%. This finding shows that the malnutrition status experienced by the subjects in this study was influenced by eating patterns that were less than the recommended percentage of RDA.

### 3.4. Effects of Consumption of Probiotic Chocolate on the Gut Microbiota

**Figure 2:**
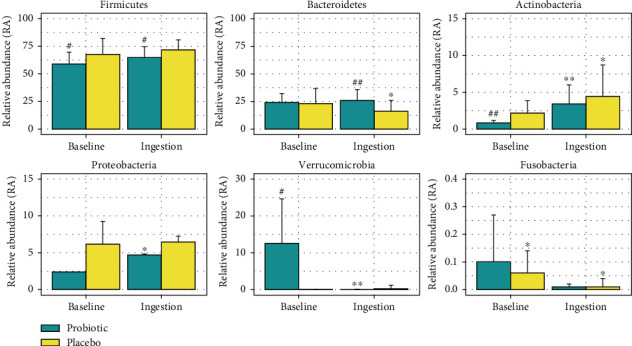
Gut microbiota composition between groups at the phylum level. Asterisks indicate differences within group comparison with baseline using the Wilcoxon signed-rank test (^∗^*p* < 0.05^∗∗^*p* < 0.01). Sharps indicate significant differences between groups using the Mann–Whitney *U*-test (#*p* < 0.05 ##*p* < 0.01).

The gut microbiota of the subjects was analyzed. As observed in the studies, the major phyla in both groups were *Firmicutes* and *Bacteroidetes* (Figure 2).

The relative abundances (RAs) of *Firmicutes* of the probiotic group were significantly lower than placebo at the baseline and after ingestion period. Based on the work of Krajmalnik-Brown et al. [26], the abundance of *Firmicutes* increased the number of lipid droplets, thus improving lipid absorption. This condition increased the energy harvest capacity by modulating calorie absorption's effectivity. It was also related to the high-sugar–low-fiber diet of the undernourished group. Bacteroidetes were found to be significantly more abundant (*p* < 0.01) in the probiotic group than placebo after ingestion. According to some studies, the most produced SCFAs by *Bacteroidetes* are acetate and propionate. *Bacteroidetes* contribute to the energy extraction efficiency of nondigestible polysaccharides. Hence, decreasing the abundance of this bacterium may cause the decreased activity of the N-glycan pathway. Therefore, maintaining the abundance of *Bacteroidetes* will be beneficial for energy production [27, 28]. Compared to the before ingestion period, the probiotic group showed increases of *Actinobacteria* of *Proteobacteria phyla*. Studies proposing mechanisms on how probiotic can influence human health showed similar result, which is to maintain optimal intestine immunity and prevent the leaky gut phenomenon by ensuring homeostasis of gut bacterial composition [29]. *Actinobacteria* and *Proteobacteria* are two major phyla found in human gut microbiome [30]. Therefore, the shift of the two phyla after probiotic administration by unhealthy subjects would be inevitable. According to Binda et al. [31], *Actinobacteria* plays a pivotal role in the maintenance of gut barrier, owing to the great production of SCFAs by one of its genus, *Bifidobacteria*. *Proteobacteria* is a commensal phylum found in the healthy gastrointestinal tract with protective functions in the immunoregulatory system [32]. However, extreme outgrowth of this phylum has been mentioned as having a correlation with dysbiosis and metabolic disorders [33]. Unfortunately, the causal effect of *Proteobacteria* abundance level in the human gut has not been clearly dissected and requires further investigation.

**Figure 3:**
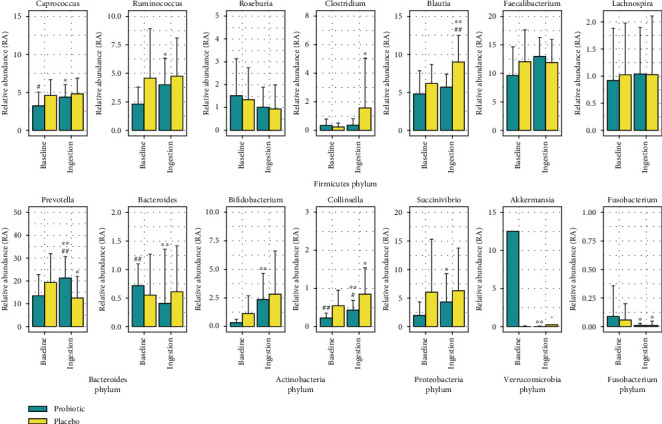
Gut microbiota composition between groups at the genus level. Asterisks indicate differences within group comparison with baseline using the Wilcoxon signed-rank test (^∗^*p* < 0.05^∗∗^*p* < 0.01). Sharps indicate significant differences between groups using the Mann–Whitney *U*-test (#*p* < 0.05; ##*p* < 0.01).

At the genus level (Figure 3), some genera, such as *Collinsella*, *Bacteroides*, *Parabacteroides*, *Prevotella*, *Blautia*, and *Akkermans*ia, showed significant differences in the RA between groups.

*Prevotella* was the most abundant microbiota at the genus level in both groups. The dominance indicates that the subjects' gut microbiota belongs to the *Prevotella* enterotypes which correlated to carbohydrate and fiber-rich diet [34]. In the probiotic group, five genera, including *Bifidobacterium*, *Collinsella*, *Bacteroides*, and *Prevotella*, and an uncharacterized genus of *Clostridiaceae*, showed significant differences (*p* < 0.01) after ingestion period. Several studies have reported the relation between these genera with beneficial health effects. The genus *Bacteroides*, alongside *Clostridium*, can convert primary bile acid to secondary bile acid [35]. The secondary bile acid can activate TGR5 receptors, which play a role in body weight, glucose metabolism, immune system modulation, and liver function [36]. *Bifidobacterium* can protect the gastrointestinal environment and is relatively common in individuals with normal body weight [37–39]. *Ruminococcus* increased significantly (*p* < 0.05) in the probiotic group during the intervention. According to Turnbaugh et al. [40], *Ruminococcus* can decompose undigested food fibers into SCFAs, such as acetic acid, propionic acid, butyric acid, and lactic acid. This genus also increases energy intake through intestinal absorption.

A previous study by Gatya et al. [41] reported that compared to children with normal nutrition status, undernourished children in Lombok, Belanting, have significantly lower *Ruminococcus*, *Bacteroides*, *Bifidobacterium*, *Collinsella*, *Succinivibrio*, and *Akkermansia*. The baseline data for the probiotics group in this study was used for the study by Gatya et al. [41] as the undernourished participants' gut microbiota composition. However, the two research were different as this study used the randomized double-blind trial with intervention of probiotic chocolate consumption; whereas the research by Gatya et al. [41] was a case-control study without intervention.

### 3.5. Effects of Consumption of Probiotic Chocolate on the Gut Microbiota Distribution

**Figure 4:**
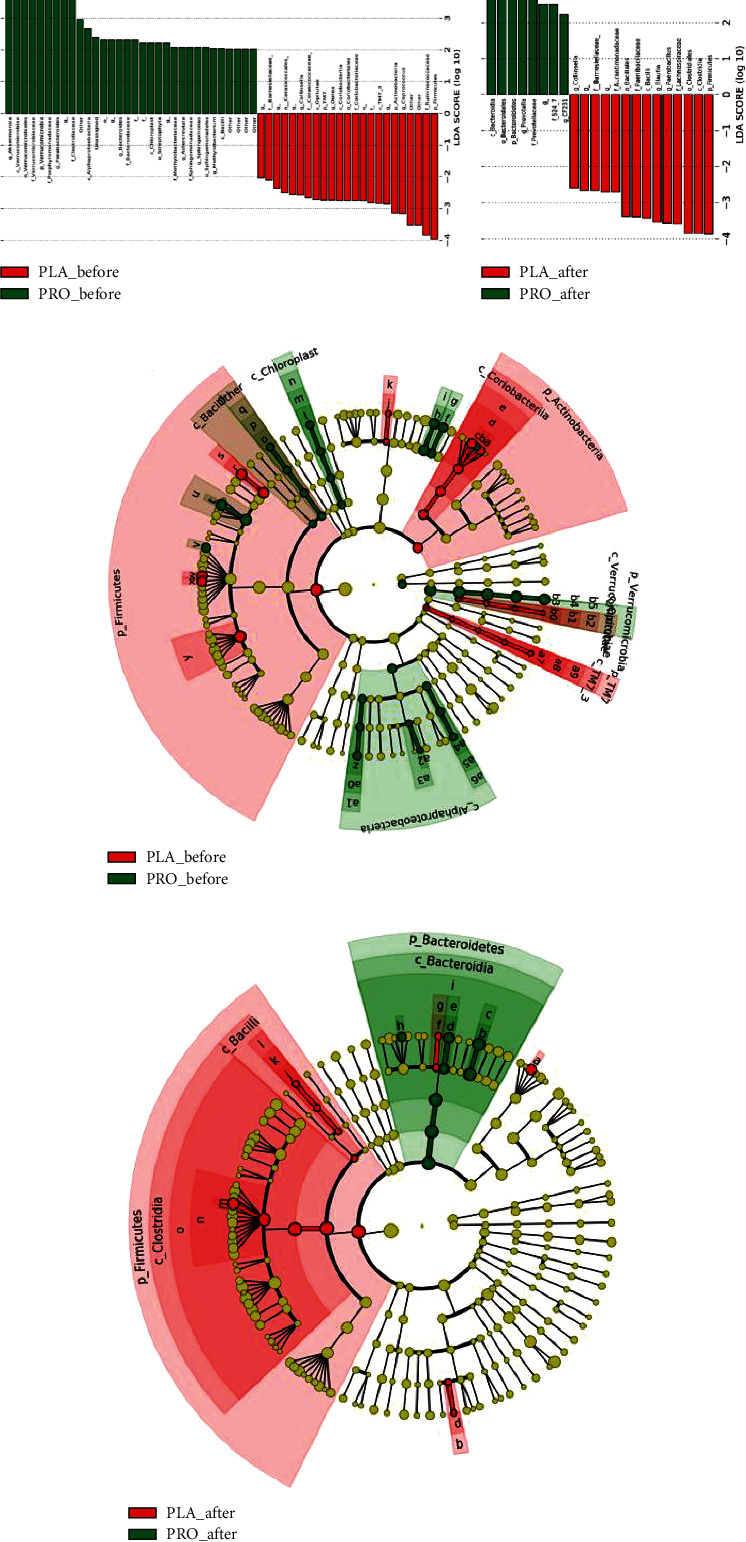
Gut microbiota taxonomic profile. The bar chart and cladogram show the taxa discrimination between the two groups based on cladogram and LDA method. Taxa with alpha value of 0.05 and LDA score of > 2.0 were considered as significant between groups (a) before ingestion and (b) after ingestion. Phylogenetic contribution of microbiota between the group (c) before ingestion and (d) after ingestion was also shown in the cladogram.

Figure 4 shows the gut microbiota taxonomic profile found in this study.

Based on the results of fecal sample analysis, 32 and 23 bacteria were significantly different in the probiotic and placebo groups before the treatment, respectively. As shown in Figure 4(a), the RAs of taxonomic groups with an LDA score greater than 10^4^ were summed for *Verrucomicrobia* and *Akkermansia* before the treatment. After the treatment, *Bacteroidetes* became more abundant, whose LDA score increased up to 10^4^. A cladogram was also generated from LEfSe, and it showed the most abundant taxa enriched in the microbiota, indicated by red and green colors for the placebo and probiotic groups, respectively. The taxonomic levels were represented by rings, and the diameter of each ring was proportional to the abundance. Before treatment, *Firmicutes* and *Actinobacteria* dominated the placebo group. After treatment, *Firmicutes* remained abundant in the placebo group, but the level of Actinobacteria decreased. Meanwhile, in the probiotic group, the phylum abundance favored *Bacteroidetes*. Overall, the gut microbiota became more heterogenous before the ingestion and slightly returned to normal and common gut microbiota composition after ingestion by the probiotic group.

**Figure 5:**
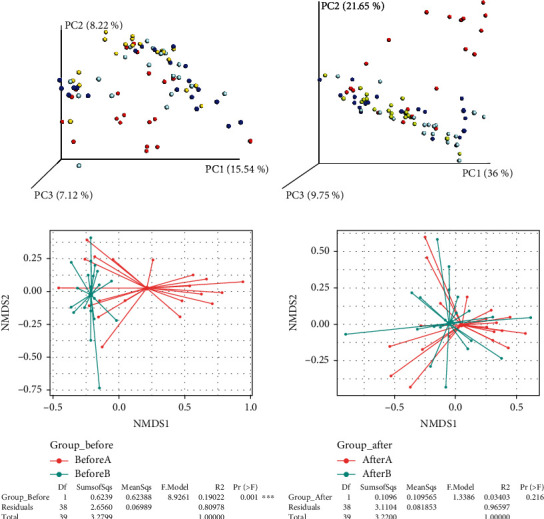
Gut microbiota beta diversity. (a) Unweighted UniFrac, (b) weighted UniFrac, (c) Bray–Curtis before ingestion, and (d) Bray–Curtis after ingestion. Legends: placebo group before ingestion (blue), placebo group after ingestion (green), probiotic group before ingestion (red), and probiotic group after ingestion (yellow).

Gut microbiota diversity was also observed using alpha and beta diversities. Beta diversity reflects the gut microbiota variation. Significant differences were observed in the *β*-diversity between groups before and after ingestion (Figure 5 for weighted UniFrac and unweighted UniFrac, Bray–Curtis dissimilarity [*p* = 0.01; *R*^2^ = 0.19022]).

### 3.6. Effects of Consumption of Probiotic Chocolate on Fecal pH

The weighted UniFrac was determined based on the OTUs' abundance, whereas the unweighted UniFrac was determined based on the phylogenetic relationship of OTUs. The analysis of NMDS was conducted based on Bray–Curtis dissimilarity to visualize the differences between groups. Group similarities were implied by the distances between data points. During calculation, the stress factor reflected the similarities between groups. A zero value of the stress factor showed great similarity. Meanwhile, a stress factor value closer to 1 indicated a poor representation. From the results, the stress factor between groups before and after ingestion was less than 0.2, thus reflecting great species similarities.

**Figure 6:**
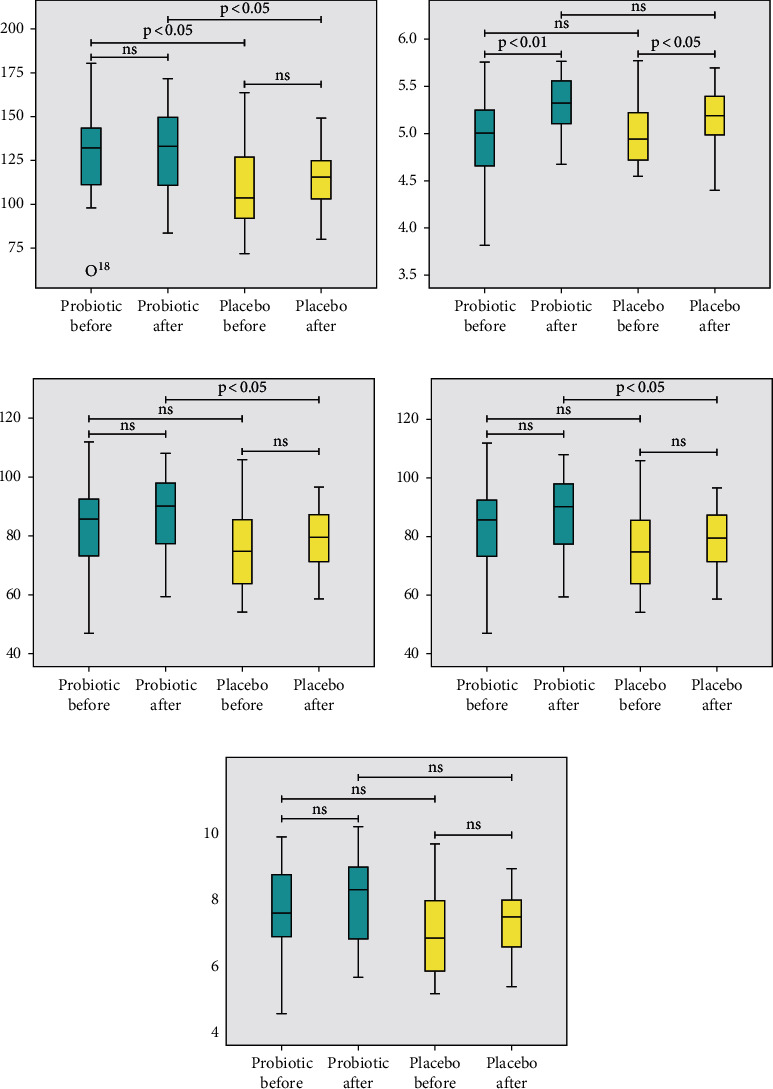
Gut microbiota alpha diversity. (a) Chao describes the species abundance between groups; (b) Shannon index shows the species diversity between groups; (c) observed OTU count among different species/OTU based on the community evenness; (d) the observed species was similar to Chao, which assumes species richness between two groups; (e) PD whole tree describes the comparison of phylogenetic diversity. Significant values within group comparison at *p* < 0.05 (Wilcoxon signed-rank test) and between group comparison at *p* < 0.05 (Mann–Whitney *U*-test).

Five metrics of alpha diversity, including Chao, Shannon, observed OTUs, PD whole tree, and observed species (Figure 6), were observed in this study.

From the results, the alpha diversity between baseline groups showed no significant difference. Thus, the gut microbiota conditions of the baseline groups were similar. Significant results for the comparison of species diversity were found in the probiotic group after the intervention. The community evenness and abundances of different species after the intervention between groups were significantly different, implying the effect of probiotic intervention. Meanwhile, no significant result was observed for the comparison of phylogenetic diversities between groups and during intervention.

### 3.7. Effects of Consumption of Probiotic Chocolate on Fecal SCFA Concentration

Table 3 shows the effect of the consumption of probiotic chocolate on SCFA concentrations, including those of acetic acid, propionate, and butyrate.

The data showed no significant changes in the SCFA within and between groups. These results are contrary to the findings of Kamil et al. [42], who stated that the consumption of a probiotic gummy containing *L. plantarum* Dad-13 (s 8.96 × 10^8^–1.16 × 10^9^ CFU/3 g) showed a significant difference in the total SFCA and butyric acid after consumption by moderately undernourished infants in Yogyakarta, Indonesia. This finding can be due to the low numbers of microbiota that synthesize SCFAs in undernourished children's colon; among such microbiota, *Bifidobacterium* can synthesize some vitamins, folic acid, and SCFAs [43]. After the intervention phase, the abundance of *Bifidobacterium* was still low compared with that in healthy children in Yogyakarta and Bali [34]; therefore, SCFAs showed no change after the consumption phase. In addition, the low consumption in undernourished children in Belanting, East Lombok, can cause no change in the value of SCFA.

### 3.8. Effects of Consumption of Probiotic Chocolate on Fecal pH

**Figure 7:**
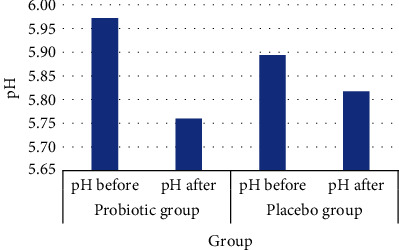
Effect of the consumption of probiotic chocolate on fecal pH of subjects in undernourished children.

Figure 7 shows a significant decrease in pH in the probiotic group (*p* = 0.001) and an insignificant decrease in the placebo group (0.077).

This finding can be due to the consumption of probiotic *Lactobacillus plantarum* Dad-13, which provided an acidic condition in the colon. This result also correlated with the increased populations of other bacteria, such as *Firmicutes*, *Bacteroidetes*, *Actinobacteria*, and *Proteobacteria*, and an insignificant increase in the level of SCFAs. A decrease in the fecal pH of subjects can be caused by a decrease in pH in the large intestine due to the organic acids, which stimulate motility in the large intestine, produced by bacteria (butyric acid, propionic acid, and lactic acid). However, changes in fecal pH are influenced by the number and activity of microorganisms in the large intestine that produce acids such as lactic acid.

## 4. Conclusions

In conclusion, a nutrient intake that is less than the RDA standard is one of the factors that affect children with undernourished nutritional status. The consumption of chocolate with *Lactobacillus plantarum* Dad-13 probiotics for 3 months does not significantly affect the weight, height, BMI, SCFA, and fecal quality of malnourished children. However, the consumption of chocolate probiotics has a positive effect on the increased abundance of beneficial gut microbiota, such as *Bacteroidetes* and *Bifidobacterium*.

## Figures and Tables

**Table 1:** The data on the characteristics of research subjects.

**Characteristics**	**Groups**	
	**Placebo (** **n** ** = 28)**	**Probiotic (** **n** ** = 28)**
Gender		
Male, *n* (%)	13 (46.43%)	13 (46.43%)
Female, *n* (%)	15 (53.57%)	15 (53.57%)
Age (year and month)	10.5	10.3
Weight (kg)	23.42 ± 2.88	23.53 ± 2.71
Height (cm)	125.79 ± 5.96	126.78 ± 5.69
BMI (kg/m^2^)	14.75 ± 0.88	14.62 ± 0.99
Food intake (per week)	4.86 ± 1.92	4.57 ± 1.88
Milk consumption (per week)	2.64 ± 1.20	2.79 ± 0.98
Cereal consumption (per week)	1.07 ± 0.37	1.07 ± 0.37
Food supplement consumption (per week)	1.07 ± 0.37	1.14 ± 0.74
Probiotic consumption (per week)	1.29 ± 0.70	1.43 ± 0.98
Prebiotic consumption (per week)	2.29 ± 1.22	2.07 ± 1.13
Alcohol consumption	No	No
Smoking	No	No
Staying up late	No	No
Stress	No	No
Gastrointestinal surgery	No	No

**Table 2:** Average nutrient intake of subjects of undernourished children during the intervention period.

**Nutrient intake**	**Groups**				**p** **value**
	**Placebo (** **n** ** = 28)**	**% RDA**	**Probiotic (** **n** ** = 28)**	**% RDA**	
Energy (kcal)	835.39 ± 402.54	46.71	741.73 ± 247.52	40.19	0.49
Protein (g)	25.73 ± 9.54	57.23	23.22 ± 7.85	48.40	0.34
Fat (g)	27.25 ± 9.98	45.15	25.86 ± 9.57	42.46	0.66
Carbohydrate (g)	111.67 ± 38.75	41.52	103.47 ± 32.89	37.65	0.43
Dietary fiber (g)	3.38 ± 1.18	13.31	2.78 ± 1.23	10.74	0.03∗
Vitamin A (*μ*g)	184.51 ± 80.93	32.86	164.43 ± 65.79	29.37	0.61
Vitamin E (mg)	2.20 ± 0.99	20.39	2.01 ± 0.85	18.67	0.73
Vitamin B1 (mg)	0.22 ± 0.09	22.58	0.21 ± 0.10	21.48	0.62
Vitamin B2 (mg)	0.33 ± 0.14	33.00	0.32 ± 0.15	31.04	0.64
Vitamin B6 (mg)	0.39 ± 0.16	35.02	0.40 ± 0.18	32.21	0.93
Folic acid (mg)	54.11 ± 18.26	15.14	44.39 ± 19.20	12.35	0.02∗
Vitamin C (mg)	11.05 ± 5.08	22.99	9.24 ± 4.65	19.35	0.17
Sodium (mg)	139.53 ± 65.81	11.14	140.95 ± 54.64	11.57	0.86
Potassium (mg)	435.20 ± 161.45	11.48	477.42 ± 211.21	12.47	0.64
Calcium (mg)	147.97 ± 66.73	13.29	129.80 ± 56.42	11.55	0.27
Magnesium (mg)	106.65 ± 40.97	69.90	89.73 ± 27.69	58.09	0.05∗
Phosphorous (mg)	319.47 ± 101.74	41.18	304.26 ± 98.73	36.46	0.54
Iron (mg)	3.38 ± 1.28	39.04	3.00 ± 1.32	35.23	0.19
Zinc (mg)	3.33 ± 1.02	51.73	3.28 ± 1.02	39.39	0.84

^*^< 0.05 represents the significance of the data between the probiotic and placebo groups (Mann–Whitney *U*-test), recommended dietary allowances (RDA), and data displayed as the mean ± standard deviation.

**Table 3:** SCFA concentration for each subject.

**SCFA**	**Group**	**Mean ± SD**	
		**Baseline**	**Ingestion**
Acetate	Probiotic	8.30 ± 2.06	8.30 ± 2.06
	Placebo	7.76 ± 3.87	7.77 ± 2.18
Propionate	Probiotic	2.13 ± 0.74	2.13 ± 0.71
	Placebo	2.38 ± 1.87	2.38 ± 1.30
Butyrate	Probiotic	1.87 ± 1.02	2.48 ± 0.69
	Placebo	1.88 ± 1.10	1.95 ± 0.77

## Data Availability

The data presented in this study are available upon request from the corresponding author. The data are not publicly available due to privacy protection.
